# The Past, Present, and Future: A Discussion of Cadaver Use in Medical and Veterinary Education

**DOI:** 10.3389/fvets.2021.720740

**Published:** 2021-11-10

**Authors:** Caitlin Varner, Lucinda Dixon, Micha C. Simons

**Affiliations:** Center for Innovation in Veterinary Education & Technology, Lincoln Memorial University College of Veterinary Medicine, Harrogate, TN, United States

**Keywords:** veterinary education, anatomy, cadavers, dissection, preservation, medical education

## Abstract

Cadaver usage in medical training, although controversial, has persisted over centuries. In veterinary education various methods have been proposed to either improve cadaver preservation, reduce cadaver use, or to replace cadavers entirely, but to date few have gained popularity. This manuscript seeks to: (i) describe the history of cadavers in medical and veterinary education; (ii) compare available cadaveric preservation methods; (iii) reflect on applications of cadaver use in the educational setting; (iv) discuss alternatives to traditional cadaver use; and (v) consider the perceptions of the stakeholders who use them.

## Introduction

Cadavers have been an important part of medical education for hundreds of years and their use remains popular in education. While controversial, the use of cadavers has been widely regarded as one of the most effective methods of learning about the body, whether it be animal or human. In this manuscript the authors review the methods of preserving cadavers, a history of their use in education, applications for cadaver usage in medical and veterinary education curricula, and proposed alternatives to cadaver use.

Multiple literature searches were performed using online databases including PubMed and Google scholar. Relevant medical education and veterinary education terms were used to search databases. Initial searches used broad terms such as “medical (or) veterinary education and cadavers.” Additional searches using more specific key terms such as a “anatomy, ” “preservation”, and “cadaver alternatives” were used to expand our search.

## The Past

### History of Cadaver Use in Medical Education

While cadavers have been integral to teaching medicine for centuries, their importance and use has evolved significantly with time. In 1542, Vesalius dissected a cadaver to compare perceived anatomical knowledge to a real body (1). His goal was to correct misinformation and to expand understanding of the human form. His use of this prosection led to the creation of a more patient centered approach to the scientific method (1). In the 18th century, Morgagni began performing dissections during autopsies in an effort to connect presenting symptoms with the pathology found in the body post-mortem, leading to a more complete understanding of various afflictions and how they affect the body. It also allowed the introduction of more thorough diagnostics at the time (1).

The use of cadavers proved to be an important step forward for the medical community, and their use continued to expand until the 1980s when medical school reforms began and cadaver usage decreased due to a shift in focus toward problem solving and case study examples (2). This reform still impacts medical education, and the modern student has experienced a greater than fifty percent decrease in cadaver use than what was previously common (2). Despite this decline, dissections are still used as a foundation of medical training in undergraduate, graduate, and surgical training programs throughout Africa, the United States, and Canada (2). Having experience with dissection in medical science and/or autopsy related courses allows practitioners to better understand how the body works and how certain presentations or symptoms can manifest (2). Since the decline of cadaver dissection over the past 20 years, medical training worldwide has noted lower proficiency in anatomy and lower levels of surgical skills in graduating medical students (2).

### Applications for Cadavers in the Veterinary Curriculum

There is a paucity of literature regarding the history of cadaver use in veterinary education specifically, so a direct comparison between medical and veterinary training programs is challenging. However, given the similarity of the programs it is not unreasonable to speculate that the considerations and perceptions of cadaver use are similar. Although sourcing and using animal cadavers has fewer ethical issues than human cadavers, the use of cadavers in veterinary education is not without challenge. These challenges include the potential for infectious agents, exposure to chemical fixatives, sourcing cadaver material, and the expense of collection and storage of specimens, as well as the ethical questions raised by the procurement and use of cadavers in veterinary medicine (3).

Anecdotally, veterinary education is still heavily reliant on cadavers in anatomical training. Additional uses for cadavers in veterinary medicine include clinical skills laboratories for hands-on technical skills practice, and research (both educational and clinical). Educators continue to seek ways to minimize or replace cadaver usage; common methods include raising the number of students assigned to a single cadaver, increasing the use of prosections, and utilizing alternative educational technologies including virtual dissection tables (4). Veterinary students perceive anatomy to be an important preparatory step for their clinical endeavors and report enjoying the ability to practice clinical techniques on cadaveric specimens (3) so removal of cadavers from the curriculum would need to be carefully considered and robust alternatives implemented.

## The Present

### Cadaver Preservation and Impact on Use

Over time, cadavers have been embalmed in many ways to ensure they are appropriate for dissection and long-term storage. Availability and expense are also important considerations in an education setting due to the large number of cadavers needed. Although the most common preservation method is currently formaldehyde based, this method is not ideal because of the health risks common with formaldehyde, along with the inability for the cadavers to remain life-like. Formaldehyde preservation methods show low anti-fungal activity which can lead to health concerns if the cadavers cannot properly be preserved. These solutions are also highly toxic, having been found to be carcinogenic, irritate mucous membranes, and have a neuropsychological effect if there is too much exposure (5). Formaldehyde-treated cadavers also show high rigidity, making them difficult to move and less life-like. These negative side effects of using formaldehyde as a preservation method have led to the search for a healthier alternative.

Alternatives to formaldehyde-based formulas have been tested, although none have become popular to date. One common alternative being considered is the Thiel embalmed cadaver (TEC), a method that combines salts and reduced levels of formaldehyde and formalin for fixation. A study directly comparing TEC with formalin embalmed cadavers (FEC) asked undergraduate and postgraduate students their opinions of each as they were using them. Students who have never seen or worked with a cadaver preferred the FEC over the TEC because it was easier to process emotionally and easier to identify structures *via* pictures from textbooks (6). Postgraduate students, especially those trying to learn surgical methods, tended to prefer TEC over FEC due to the improved smell, more life-like appearance, but there was no difference noted in the user's emotional response to the activities (6). Using TEC is considered more appropriate for surgical methods because the body is less rigid and moves in a more life-like manner, giving a more realistic comparison to live surgery (6).

Another developing method of embalming is nitrite pickling, which is comparable to formaldehyde in its ability to preserve the body with low microbial growth (7). In this process, nitrite pickling salt is mixed with ethanol and Pluriol^Ⓡ^ (a proprietary blend of polyethylene glycols) to preserve the cadaver. Janczyk et al. investigated the nitrite pickling method in cadavers with open abdomens and those with closed abdomens. These cadavers were compared to 20 cadavers preserved with formaldehyde. All 40 cadavers were monitored over 12 months throughout dissections. The open abdomen cadavers remained suitable throughout with periodic treatments of ethanol and Pluriol, and after 30 weeks only one species of microbe was recorded. The closed abdomen cadavers did not perform as well. Although there was no signs of autolysis, loss of natural features was reported and five species of microbes were recorded after 24 weeks. The formaldehyde group showed high numbers of mold colonies present and seven different microbe species found throughout the cadavers. The authors concluded that nitrite pickling can properly preserve a cadaver in a healthy, safe manner, though it is highly improved if the abdomen remains open (7).

A third method for preserving cadavers is a saturated salt solution. This method uses a continuous perfusion of a saturated salt solution for 6–8 hours and then stores the cadaver immersed in a saturated salt solution (8). This method negatively affected the color and texture of the specimens, though all vessels remained intact and useful. Saturated salt solution preservation is cost effective and can be maintained for long periods of time; the cadaver also remains visually similar to a formalin-fixed cadaver, but does not present as many health concerns. As a result, the saturated salt fixation is becoming increasingly popular (8).

The modified Larssen solution is an alternative preservation method that uses a combination of 10% formalin, glycerol, chloral hydrate, sodium sulfate, sodium bicarbonate, sodium chloride, and distilled water. This mixture is then diluted using one part solution and three parts distilled water. The cadavers are washed and the solution is injected into the carotid cannula. After fixation and between uses of the cadaver, it is stored in a plastic bag hanging in a walk-in freezer for up to 4 months. Before use, the cadaver is thawed in water tanks for 24 hours (9). In a study conducted by Da Silva et al., each cadaver was used four times and never emitted odors while maintaining texture, color, and consistency of skin and muscles similar to live animals. However, the oral mucosa became pale over time on five of 97 cadavers, but none had joint movement loss and could easily be manipulated. By the 4th week, 14 cadavers had desquamation in the abdominal and inguinal regions but this did not impact instruction because those areas were not used (9). A later study used this same modified Larssen solution and an identical protocol but looked at the effects of administering heparin immediately after euthanasia. In this study 14 dog cadavers were divided into two groups, with and without heparin treatment, with seven cadavers each (10). These groups were ultimately found to have no statistically significant difference in viscera quality, mobility, color, or odor after use by surgical instruction teams (10).

In summary, studies support both the continued use of cadavers for anatomic and surgical training and the search for alternative preservation methods that are safe and effective in human and veterinary medical education. Preservation methods are evolving in an effort to improve cadaver quality and minimize the number of cadavers required. While formaldehyde based solutions remain the most common, despite many concerns regarding their use, alternatives such as TEC, nitrile pickling, saturated salt solution, and Larssen solution are growing in popularity. However, a single formaldehyde alternative has not yet emerged as superior or the new standard.

### Alternative Methods and Their Effectiveness

As the search continues for the best way to teach subjects like anatomy and surgical skills, alternative approaches to cadaver use in veterinary education are being investigated and typically fall into the following categories: living or applied anatomy, education supported by medical-imaging, models and simulators, and/or the use of virtual reality platforms. Although growing, there remains little evidence on the educational impact of teaching with or without cadavers (11).

Models and simulators come in many forms and can be used to augment instruction throughout students' training. High fidelity models, like the Canine Syndaver™, are intriguing as cadaver replacements. While they effectively replicate basic anatomy and can be used for some procedural practice, issues relating to storage space, required maintenance, and expense limit their use at this time.

Three-dimensional (3-D) digital and printed models of different areas of the body are increasingly popular, allowing students to manipulate views and get a clean and full understanding of how different tissues work together. Such models have also been used to help guide a cadaver dissection (12) and allow educators to move away from the complexity and risks of cadaver dissection. Models do not present the same health and mental well-being risks, and they present the body in a cleaner, easier to understand way (12). Models can be especially useful when explaining relationships in structures that are too small to see in a cadaver (12). Both digital and physical 3-D models are used to enhance education as a lower cost comparison to cadavers (12). When comparing 3-D printed models to cadavers directly, a meta-analysis found that test results from students using 3-D models were higher than students using traditional cadaver specimens to learn (13).

Computer assisted learning (CAL) programs have increasingly been used as a solution for concerns regarding cadaveric dissection in veterinary education (14–16). Such technology has additional benefits, including accessibility, convenience, and ease of collaboration via various digital devices. However, the time to develop such programs and cost of user licensing can create a barrier to large scale implementation. One such program is IVALA^Ⓡ^, a 3-D virtual anatomy program accessible from a student's computer that allows interaction with a virtual canine specimen. Using this program students can identify, move, rotate, magnify, and remove anatomic structures while getting a description of each structure as they interact with it. Studies by Little et al. found that students who used the program scored significantly higher on their examinations compared those who did not (14, 15). Student users reported a beneficial and enjoyable attitude toward the program but did not support replacing cadaveric dissection completely with the program or any other form of CAL (15). In subsequent studies the same authors propose that certain disciplines (e.g., cardiology) may benefit more from the use of augmented reality platforms (16). These preliminary results of CAL are encouraging, and further research is needed to explore and define the ideal use of this technology in veterinary education.

A meta-analysis investigating the effectiveness of CAL and similar technologies in medical education found that their use resulted in higher factual knowledge, better spatial knowledge acquisition, and increases in learners' perception of effectiveness when compared to other methods of anatomy instruction (17). However, additional studies suggest that while such technologies are indeed helpful, their implementation does not negate or remove the need to use cadavers within the curriculum. Instead, virtual or augmented reality programs may be best used in conjunction with traditional methods and other radiology modalities rather than as stand-alone tools at this time (18, 19).

### Student Perceptions of Cadavers and Alternatives

While educators debate the extent to which cadavers should be used in teaching physicians and veterinarians, the student perception data is less contentious. In one study, the majority of medical students feel that cadaver dissection was more conducive for learning anatomy when compared to virtual software programs. These students saw cadaver dissection as vital to their success in learning anatomy and were excited to perform their first dissection with guidance from an instructor (20). Other studies in medical education found both students and faculty to be in favor of access to cadaveric specimens and supportive of more traditional methods of small group teaching for anatomy based learning (18, 19, 21). These students and faculty also perceived other teaching methods, including e-learning, anatomical models and surgical videos, as useful educational tools (18, 19, 21).

Studies from the veterinary literature demonstrate similar findings (9, 15, 22). Ghosh et al. found that Turkish veterinary students thought the use of cadavers was absolutely necessary in their anatomy education (22). Although provided with a variety of study alternatives, including books, plastic models, and computer models, the cadaver was considered the most useful resource, with the other resources viewed as supplemental (22). North American veterinary students also reported that cadaveric dissection was the most useful teaching modality of learning anatomy, followed by 3-D computer simulation, with a tie between textbooks and lecture (15).

### The Best of Both Worlds?

Both students and educators support the synergistic use of cadavers, virtual reality, and the integration of clinically relevant materials over the complete removal of cadavers from the curriculum. Combined educational approaches allow for effective delivery of anatomical content and integration of clinical skills with a hands-on experience (22, 23). Simpson described a combined educational approach where an anatomy course used a single cadaver over the duration of the semester, alongside various radiology modalities and problem-based learning modules. Over a 10-year period, student reviews of the class reported higher positive attitudes and improved perception of learning in this course compared to traditional anatomy instruction (23). This method was reported as a viable, cost-effective alternative to the traditional, cadaver-based approach (23).

## The Future

### Future Directions

There are undoubtably challenges with the use of cadavers in both medical and veterinary education, although the academic benefit may outweigh these issues at this time (17–24). However, as alternatives to cadaveric use continue to improve, the use of cadavers may continue to decline. Additional research on cadaver alternatives is necessary to explore and evaluate the suitability of implementing these alternatives and to measure the educational outcomes associated with their use. Student perceptions and acceptance of these new, integrated, and multimodal teaching approaches should also be considered.

## Conclusion

Although cadaver use in veterinary and human medical training has declined over the last few decades, a complete replacement for cadaver use in developing anatomic and surgical skills using cadavers does not appear to exist at this time. Continued improvement of embalming methods may help to improve cadaver quality and longevity, ensuring that the cadavers are as life-like and safe to use as possible. Concurrently, technological advances including virtual reality continue to evolve and may eventually become a suitable replacement for cadavers. The search for alternative anatomy and clinical skills training methods will undoubtably continue, but at present, cadaver dissection continues to be an integral part of human and veterinary medical training. Cadaver use should be augmented by supplementary teaching modalities to provide the best learner experience.

## Author Contributions

CV participated in the literature review, writing, and editing for the manuscript. LD participated in the writing and editing for the manuscript. MS participated in the conception of the study, literature review, writing, and editing for the manuscript. All authors contributed to the article and approved the submitted version.

## Funding

This study was funded by LMU-CVM Intramural Grant.

## Conflict of Interest

The authors declare that the research was conducted in the absence of any commercial or financial relationships that could be construed as a potential conflict of interest.

## Publisher's Note

All claims expressed in this article are solely those of the authors and do not necessarily represent those of their affiliated organizations, or those of the publisher, the editors and the reviewers. Any product that may be evaluated in this article, or claim that may be made by its manufacturer, is not guaranteed or endorsed by the publisher.
